# Field-Induced Single Molecule Magnets of Phosphine- and Arsine-Oxides

**DOI:** 10.3389/fchem.2018.00420

**Published:** 2018-09-12

**Authors:** Matilde Fondo, Julio Corredoira-Vázquez, Ana M. García-Deibe, Jesús Sanmartín-Matalobos, Juan Manuel Herrera, Enrique Colacio

**Affiliations:** ^1^Departamento de Química Inorgánica, Facultade de Química, Universidade de Santiago de Compostela, Santiago de Compostela, Spain; ^2^Departamento de Química Inorgánica, Facultad de Ciencias, Universidad de Granada, Granada, Spain

**Keywords:** terbium, dysprosium, triphenylarsine oxide, phosphine oxide, single ion magnet

## Abstract

The coordination chemistry of dysprosium and terbium toward phosphine and arsine oxides was further explored. Thus, the new nitrate [M(NO_3_)_3_(Ph_3_PO)_3_] (*M* = Tb, **1**; Dy, **2**), [Dy(NO_3_)_3_(EtOH)(Ph_3_XO)_2_] (*X* = P, **3**; As, **4**), chloride [DyCl_2_(Ph_3_AsO)_4_]Cl (**5**), triflate [Dy(OTf)_2_(MePh_2_PO)_4_]OTf (**6**; OTf = triflate) and hexafluoroacetylacetonate [M(hfa)_3_(Ph_3_PO)_2_] (hfa = hexafluoroacetylacetonate; *M* = Tb, **7**; Dy, **8**) complexes were isolated and fully characterized. The crystal structures of **1**·CH_3_CN, **2**·CH_3_CN, **4**, **5**·2.75EtOH·1.25H_2_O, **6**, **7**, and **8** show MO_9_ cores in **1, 2**, and **4**, with highly distorted geometry, between spherical capped square antiprism and muffin-like, hexacoordinated environments for the dysprosium ions in **5** and **6**, with octahedral geometry, and octa-coordination for the lanthanoid metals in **7** and **8**, with geometry closer to square antiprism. Comparison of the magnetic behavior of all the complexes allows analyzing which metal ion (Tb or Dy), phosphine or arsine oxide, or anionic ligand favor more the slow relaxation of the magnetization. Alternating current magnetic measurements show that only **2**, **4**, and **8** present slow relaxation of the magnetization in the presence of an external magnetic field, **8** being the complex with the highest *U*_eff_ (44.85 K) of those described herein.

## Introduction

The observation for the first time of slow magnetic relaxation in mononuclear lanthanoid complexes (TBA)[Pc_2_Ln] (TBA = ${\text{Bu}}_{4}^{\text{t}}$N^+^; Pc = phthalocyanide; Ln^III^ = Tb or Dy) (Ishikawa et al., 2003) provided a real breakthrough in molecular magnetism, opening the field of single ion magnets (SIMs) in 2003. This field has received growing attention since its origins, given that the non-trivial memory effect and quantum character in SIMs renders them as potential ultra-high density data storage medium and spintronic devices (Woodruff et al., 2013; Shiddiq et al., 2016; Lu et al., 2017). Accordingly, the huge amount of work devoted to this research during the last 15 years has led to many remarkable advances. Thus, Rinehart and Long published in 2011 (Rinehart and Long, 2011) a benchmark study where they provided a clear explanation of how the electronic structure of f-elements can in theory be manipulated to create new single molecule magnets (SMMs). In this study, they give the relationship between the coordination environment of lanthanoid ions and the magnetic anisotropy of the complex and, therefore, they suggest that one can match an appropriate ligand field to maximize magnetic anisotropy on the basis of the shapes (oblate or prolate) of the 4f-shell electron density distributions. For the oblate Tb(III) and Dy(III) ions, theoretical calculations established that the optimum environment to maximize the anisotropy is the axial one (Ungur and Chibotaru, 2011). Thus, the optimum geometry will be lineal but the coordination number two seems too low to be stabilized by the lanthanoid ions. Accordingly, in the absence of this possibility, the coordination number 7, with pentagonal bipyramidal geometry, was the most explored one.

On the basis of these starting theoretical studies, much experimental work was done and continuous attainments relating to anisotropic energy barriers (*U*_eff_) and blocking temperatures (*T*_B_) were achieved in the field of SIMs. Hence, the highest *U*_eff_ described up to now is 1815 K (Ding et al., 2016), a value significantly higher than 331 K reported for the first SIM (${\text{Bu}}_{4}^{\text{t}}$N)[Tb(Pc)_2_] (Ishikawa et al., 2003). Besides, the blocking temperatures have been continuously increasing, from the initial 1.7 K (Ishikawa et al., 2005) thorough the 20 K achieved in 2016 for an-air stable [Dy(Cy_3_PO)_2_(H_2_O)_5_]Br_3_ complex (Chen et al., 2016) to the astonishing 60 K recently reported for the metallocene complex [(Cp^ttt^)_2_Dy][B(C_6_F_5_)_4_] (Goodwin et al., 2017; Guo et al., 2017).

In spite of these advances, it should be noted that most of the SIMs with high *U*_eff_ and/or *T*_B_ are air-unstable, and this is a handicap that must be surpassed. It must be noted that a series of simple, easy to obtain, and air stable phosphine oxide complexes [Dy(R_3_PO)_2_(H_2_O)_5_]X_3_ (R = Cy_3_ or CyPh_2_, X = Cl, Br or I) show blocking temperatures between 19 and 20 K (Chen et al., 2016, 2017), the largest ones among SMMs if the 60 K reported for the unstable [(Cp^ttt^)_2_Dy][B(C_6_F_5_)_4_] is excluded. Accordingly, it seems that the coordination chemistry of lanthanoids with phosphine oxides is really interesting from the magnetic point of view. This coordination chemistry has been extensively studied, mainly by Platt (2017), but, in spite of this, the magnetic behavior of these compounds is still poorly investigated. Therefore, taking into account all the above considerations, we have decided to revise and extent the study of the coordination chemistry of Dy and Tb with phosphine and arsine oxides, and to investigate the magnetic behavior of the obtained compounds. The comparison of the magnetic properties of the isolated complexes between them allows establishing some patterns.

## Materials and methods

### General

All chemical reagents and solvents were purchased from commercial sources and used as received without further purification. Elemental analyses of C, H and N were recorded on a Carlo Erba EA 1108 analyzer. Infrared spectra were performed in the range 4000–500 cm^−1^ on a Varian 670 FT/IR spectrophotometer in the ATR mode.

### Syntheses of the complexes

**[Tb(NO**_3_**)**_3_**(Ph**_3_**PO)**_3_**] (1)**: To a solution of Tb(NO_3_)_3_·5H_2_O (0.130 g, 0.30 mmol) in acetonitrile (10 mL), triphenylphosphine oxide, (0.167 g, 0.60 mmol) and acetonitrile (10 mL) were added. The mixture was stirred for 4 h at room temperature and the resultant colorless solution was left to slowly evaporate, until single crystals of **1**·CH_3_CN precipitated. The single crystals were filtered, and they lose the acetonitrile solvate on drying to yield **1**. Yield (based on Ph_3_PO): 0.22 g (93%). M.W.: 1179.80. Anal. calcd. for C_54_H_45_TbN_3_O_12_P_3_: C 54.97, H 3.84, N 3.56%. Found: C 54.89, H 3.59, N 3.95%. IR (ATR, $\tilde{\nu }$/cm^−1^): 1120, 1153 (P = O), 1305 (${\text{NO}}_{3}^{-}$).

The same product is isolated when Tb(NO_3_)_3_·5H_2_O and Ph_3_PO are mixed in 1:3 molar ratio in acetonitrile.

**[Dy(NO**_3_**)**_3_**(Ph**_3_**PO)**_3_**] (2)** was obtained in a similar way to **1**: amounts of Dy(NO_3_)_3_·6H_2_O (0.23 g, 0.66 mmol) and Ph_3_PO (0.360 g, 1.32 mmol). Single crystals of **2**·CH_3_CN were isolated in the same way as those of **1**·CH_3_CN, which lose the acetonitrile solvate on drying to yield **2**. Yield (based on Ph_3_PO): 0.403 g (77%). M.W.: 1183.37. Anal. calcd. for C_54_H_45_DyN_3_O_12_P_3_: C 54.81, H 3.83, N 3.55%. Found: C 54.84, H 3.40, N 3.53%. IR (ATR, $\tilde{\nu }$/cm^−1^): 1120, 1153 (P = O), 1305 (${\text{NO}}_{3}^{-}$).

The same product is isolated when Dy(NO_3_)_3_·6H_2_O and Ph_3_PO are mixed in 1:3 molar ratio in acetonitrile.

**[Dy(NO**_3_**)**_3_**(EtOH)(Ph**_3_**PO)**_2_**] (3):** A hot solution of Dy(NO_3_)_3_·6H_2_O (0.137 g, 0.30 mmol) in ethanol (5 mL) was added to a hot solution of triphenylphosphine oxide (0.170 g, 0.60 mmol) in ethanol (5 mL). The mixture was stirred at room temperature for 24 h, when a colorless powder precipitated. The solid was filtered and dried in air. Yield: 0.250 g (88%). M.W.: 951.15. Anal. calcd. for C_38_H_36_DyN_3_O_12_P_2_: C 47.98, H 3.81, N 4.42%. Found: C 47.90, H 3.81, N 4.29%. IR (ATR, $\tilde{\nu }$/cm^−1^): 1119, 1150 (P = O), 1308 (${\text{NO}}_{3}^{-}$), 3346 (OH).

**[Dy(NO**_3_**)**_3_**(EtOH)(Ph**_3_**AsO)**_2_**] (4)** was obtained in the same way as **3**: amounts of Dy(NO_3_)_3_·6H_2_O (0.23 g, 0.66 mmol) and Ph_3_AsO (0.425 g, 1.32 mmol). The mixture yields a solution that by slow evaporation gives rise to single crystals of **4**. Yield: 0.313 g (46%). M.W.: 1039.05. Anal. calcd. for C_38_H_36_DyN_3_O_12_As_2_: C 43.92, H 3.49, N 4.04%. Found: C 44.44, H 3.35, N 3.92%. IR (ATR, $\tilde{\nu }$/cm^−1^): 902, 924 (As = O), 1313 (${\text{NO}}_{3}^{-}$), 3334 (OH).

**[DyCl**_2_**(Ph**_3_**AsO)**_4_**]Cl**·**1.25H**_2_**O (5**·**1.25H**_2_**O):** To an ethanol (10 mL) solution of DyCl_3_·6H_2_O (0.460 g, 1.22 mmol), Ph_3_AsO (0.77 g, 2.395 mmol) was added. The mixture was stirred under reflux for 1 h and the colorless solution was concentrated in a rotaevaporator to *ca*. 2 mL, yielding an oil. After standing for 24 h, single crystals of **5**·2.75EtOH·1.25H_2_O, suitable for X-ray diffraction studies, were isolated. The crystals were filtered and dried in air, losing the ethanol solvate to give rise to **5**·1.25H_2_O. Yield (based on Ph_3_AsO): 0.74 g (78%). M.W.: 1579.93. Anal. calcd. for C_72_H_62.5_AsCl_3_DyO_5.25_: C 54.68, H 3.95%. Found: C 54.27, H 3.33%. IR (ATR, $\tilde{\nu }$/cm^−1^): 886 (As = O), 3264 (OH).

The same product is isolated when DyCl_3_·6H_2_O and Ph_3_AsO are mixed in 1:4 molar ratio in ethanol.

**[Dy(OTf)**_2_**(MePh**_2_**PO)**_4_**](OTf)**·**THF (6**·**THF):** Dy(OTf)_3_ (0.423 g, 0.694 mmol) was dissolved in THF (7 mL), and methyldiphenylphosphine oxide (0.600 g, 1.387 mmol) was added. The mixture was stirred at room temperature for 24 h, and the resultant colorless solution was concentrated in a rotaevaporator up to *ca*. 2 mL, yielding an oil. The oil was left to stand for 10 days at room temperature until single crystals of **6**·THFwere obtained. Yield: 0.250 (47%). M.W.: 1546.63. Anal. calcd. for C_59_H_60_DyF_9_O_14_P_4_S_3_: C 45.78, H 3.88, S 6.21%. Found: C 45.79, H 3.65, S 5.91%. IR (ATR, $\tilde{\nu }$/cm^−1^): 633 (δSO_3_), 1020, 1262 (SO_3_), 1156, 1220 (CF_3_), 1132 (P = O).

The same product is isolated when Dy(OTf)_3_ and MePh_2_PO are mixed in 1:4 molar ratio in THF.

**[Tb(hfa)**_3_**(Ph**_3_**PO)**_2_**] (7):** Terbium chloride hexahydrate (0.14 g, 0.36 mmol) was dissolved in distilled water (5 mL). An ethanol solution (20 mL) of hexafluoroacetylacetone (0.22 g, 1.08 mmol) was added to the aqueous solution. An ethanolic tetramethylammonium hydroxide solution (0.1 M) was added dropwise until pH 7 was reached. After stirring the mixture at room temperature for 6 h, the solvent was eliminated in a rotaevaporator. The white solid that precipitated was dissolved in methanol (30 mL), and triphenylphosphine oxide (0.10 g, 0.36 mmol) was added. The mixture was heated under reflux while stirring for 6 h. Slow evaporation of the resultant solution yields single crystals of **7**, suitable for X-ray diffraction studies. The crystals were filtered and dried in air. Yield (based on Ph_3_PO): 0.070 (29%). M.W.: 1336.65. Anal. calcd. for C_51_H_33_TbF_18_O_8_P_2_: C 45.83, H 2.49%. Found: C 45.79, H 2.42%. IR (ATR, $\tilde{\nu }$/cm^−1^): 1138 (P = O), 1650 (C = O), 1250, 1160 (CF_3_).

The same product is isolated when TbCl_3_·6H_2_O, hfah and Ph_3_PO are mixed in 1:3:2 molar ratio in water/ethanol.

**[Dy(hfa)**_3_**(Ph**_3_**PO)**_2_**] (8)** was obtained in a similar way to **7**: amounts of DyCl_3_·6H_2_O (0.400 g, 1.07 mmol), Ph_3_PO (0.298, 1.07 mmol) and hfah (0.664 g, 3.21 mmol). Single crystals of **8** are isolated in the same way as those of **7**. Yield 0.210 (29%). M.W.: 1340.22. Anal. calcd. for C_51_H_33_DyF_18_O_8_P_2_: C 45.70, H 2.48%. Found: C 45.38, H 2.86%. IR (ATR, $\tilde{\nu }$/cm^−1^): 1136 (P = O), 1652 (C = O), 1250, 1161(CF_3_).

The same product is isolated when DyCl_3_·6H_2_O, hfah and Ph_3_PO are mixed in 1:3:2 molar ratio in water/ethanol.

### Magnetic measurements

Magnetic susceptibility direct current (*dc*) and alternating current (*ac*) measurements for **1**–**8** were performed with a Quantum Design SQUID MPMS-XL-5 susceptometer. The magnetic susceptibility *dc* data were recorded at temperatures ranging from 2 to 300 K, under a magnetic field of 1000 Oe. Magnetization measurements under magnetic fields of 0–50000 Oe at 2.0 K were also recorded. Diamagnetic corrections were estimated from Pascal's Tables. *ac* susceptibility data were registered with an oscillating *ac* field of 3.5 Oe and *ac* frequency of 1400 Hz, under diverse applied static fields (H_dc_ = 0 or 1,000) for all the reported compounds. In the case of **2** and **8**, *ac* susceptibility measurements were moreover recorded under a *dc* field of 1000 Oe at *ac* frequencies in the range 50–1400 Hz.

### Crystallographic refinement and structure solution

Crystal data and details of refinement are given in Table S1. Single crystals of **1**·CH_3_CN, **2**·CH_3_CN, **4**, **5**·2.75EtOH·1.25H_2_O, **6**·THF, **7** and **8** were obtained as detailed above. Data were collected at 100 K on a Bruker Kappa APEXII CCD diffractometer, employing graphite monochromated Mo-kα (λ = 0.71073 Å) radiation. Multi-scan absorption corrections were applied using SADABS (Blessing, 1995; Krause et al., 2015). The structures were solved by standard direct methods, using SHELXT (Sheldrick, 2015a), and then refined by full-matrix least-squares techniques on *F*^2^, using the program package SHELXL (Sheldrick, 2015b) from the program package SHELX (Sheldrick, 2008). All non-hydrogen atoms were refined anisotropically, with the exception of some atoms with low occupation sites. Most of the hydrogen atoms were included in the structure factor calculations in geometrically idealized positions. However, non-disordered hydrogen atoms, which could be potentially involved in H-bonding schemes, were mostly located in the corresponding Fourier maps. These H atoms were freely refined, or with thermal parameters derived from their parent atoms.

CCDC 1850933–1850938 and 1851725 contain the supplementary crystallographic data for this paper. These data can be obtained free of charge from The Cambridge Crystallographic Data Center via www.ccdc.cam.ac.uk/structures.

## Results and discussion

### Synthesis and spectroscopic characterization

As previously stated, the chemistry of lanthanoids with phosphine oxides is quite well studied and many complexes with different metal ion:phosphine oxide ratios were isolated (Platt, 2017). These previous studies show that the stoichiometry of the complexes seems to be dependent on the nature of the metal salt employed, and on the molar quantities of salt and phosphine oxide in the reaction mixture, but also on the cone angle of the phosphine (Tolman, 1977). Recently, it has been reported that complexes of formulas [Ln(Cy_3_PO)_2_(H_2_O)_5_]X_3_ (Ln = La, Dy, Er, Yb, Lu; X = Cl or Br) (Lees and Platt, 2014, 2015) can be easily obtained. These complexes have pentagonal bipyramidal geometry, and some of them are air stable single ion magnets, with high blocking temperatures (Chen et al., 2016). Nevertheless, similar pentagonal bipyramidal complexes were not reported for Ph_3_PO, what would lead to evaluate the influence of the aromatic *vs*. the aliphatic ring in the magnetic behavior of the complexes. With these considerations in mind, different dysprosium and/or terbium salts were initially mixed with Ph_3_XO (X = P or As) or MePh_2_PO in 1:2 molar ratio, with the intention of isolating complexes of stoichiometry Ln(R_3_XO)_2_Y_5_ (R_3_ = Ph_3_ or MePh_2_; X = P or As; Y = OH_2_ and/or monodentate anion).

**Figure 1:**
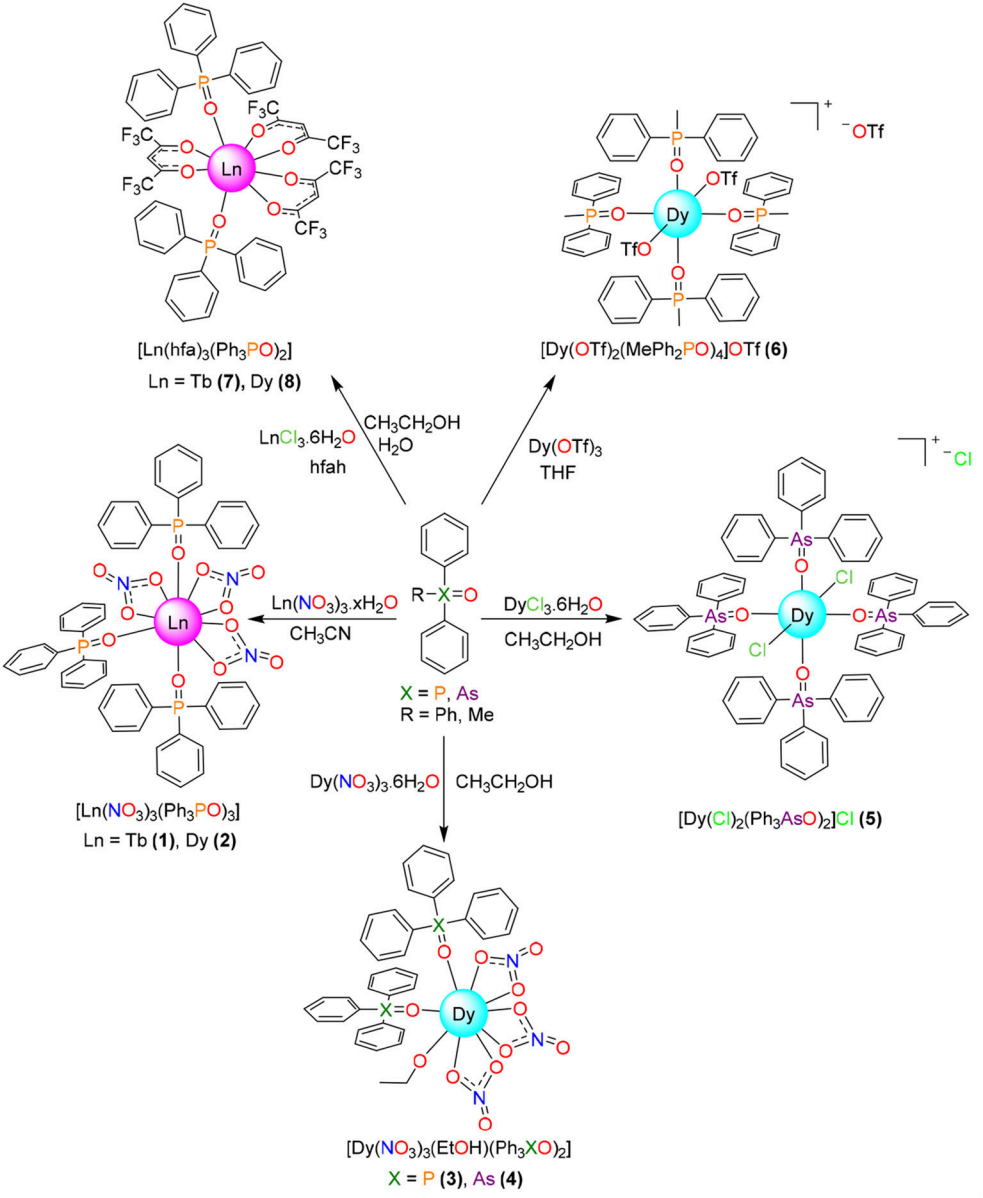
Reaction scheme for isolation of the metal complexes.

The obtained results indicate that the salt:R_3_XO molar ratio plays a secondary role in the stoichiometry of the isolated complexes (Figure 1), and that in this case the obtaining of compounds with coordination number 7 and pentagonal bipyramidal geometry was not possible. Thus, mixing of Ln(NO_3_)_2_·6H_2_O (Ln = Dy or Tb) with Ph_3_XO (X = P or As) in 1:2 molar ratio leads to isolate [Ln(NO_3_)_3_(Ph_3_PO)_3_] or [Dy(NO_3_)_3_(EtOH)(Ph_3_XO)_2_] compounds, as a function of the solvent employed in the reaction, as previously described for Ph_3_PO (Cousins and Hart, 1967). Accordingly, it also seems that the nature of the X atom (P or As) in the Ph_3_XO donor does not play any role in the stoichiometry of the isolated complexes. Besides, it should be noted that the compounds [Ln(NO_3_)_3_(Ph_3_PO)_3_] **1** and **2** are also obtained when the salts and oxides are mixed in 1:3 molar ratio, and that these complexes present the same stoichiometry as those reported for Ln = La, Nd, Eu, Er, Tm, and Yb with Cy_3_PO (Hunter et al., 2007).

When the nitrate salt was changed by dysprosium chloride, and Ph_3_AsO was added in 1:2 molar ratio, the hydrated complex [DyCl_2_(Ph_3_AsO)_4_]Cl·1.25H_2_O was obtained (Figure 1). Its stoichiometry is the same described for [DyCl_2_(Ph_3_PO)_4_]Cl·2H_2_O (Glazier et al. 2004), obtained by mixing of DyCl_3_ and Ph_3_PO in 1:4 molar ratio, but clearly differs from that of [Dy(Cy_3_PO)_2_(H_2_O)_5_]Cl_3_ (Lees and Platt, 2014), which also has been obtained by mixing of DyCl_3_ and Cy_3_PO in 1:4 molar ratio in ethanol. Accordingly, comparison of this experiment with the reactions with dysprosium nitrate and with previous results (Glazier et al., 2004; Hunter et al., 2007; Lees and Platt, 2014) clearly show that the cone angle of the phosphine plays a fundamental role in the stoichiometry of phosphine or arsine oxide complexes, but that the similarity between the chemistry of Cy_3_PO and Ph_3_XO also depends on the nature of the anion of the salt employed.

Since three nitrate donors or two of the chloride anions are coordinated to the lanthanoid metal center in **1**-**4** and related complexes (Glazier et al., 2004; Hunter et al., 2007; Bowden et al., 2014), a salt of a non-coordinating anion, such as triflate, was chosen, in order to see the influence of the coordinating ability of the anion of the salt in the final stoichiometry, and in the hope of isolating complexes of stoichiometry [Dy(R_3_XO)_2_(H_2_O)_5_]Y_3_. In this way, mixing of dysprosium triflate with MePh_2_PO in 1:2 molar ratio, and in a non-dried non-coordinating solvent such as THF, yields [Dy(OTf)_2_(MePh_2_PO)_4_]OTf, which also has a molar ratio metal:oxide of 1:4. Thus, this experiment shows that, in spite of the quantity of water present in the non-dried THF solvent, the dysprosium atom links to two poorly coordinating triflate ions, preventing the coordination of water to the metal center. Accordingly, this result resembles in part those obtained for Ce, Nd and Lu triflate complexes of Ph_3_PO (Fawcett et al., 2002; Berthet et al., 2003;), where the stoichiometry is the same, but for the Ce and Nd derivatives the coordination number is 7, with one triflate acting as a bidentate chelate ligand and the other one as a monodentate donor.

Finally, the reactivity of Dy and Tb toward Ph_3_PO, in 1:1 molar ratio and in the presence of hexafluoroacetylacetone, was studied, given that a [Eu(tmh)_3_(RPh_3_PO)] (tmh = 2,2,6,6-tetramethylheptane-3,5-dione, R = H, *m*-Me or *p*-Me) complex, with coordination number seven, was previously reported (Yanagisawa et al., 2017). As Eu^3+^ is significantly bigger than Tb^3+^ and Dy^3+^, the isolation of Tb and Dy complexes with coordination number 7 was expected even in the presence of the less sterically hindered hexafluoroacetylacetone donor. Nevertheless, this synthesis leads to complexes [M(hfa)_3_(Ph_3_PO)_2_] (M = Tb, Dy) where the metal:oxide ratio is 1:2, as it occurs in the previously described [Eu(hfa)_3_(Ph_3_PO)_2_] and related compounds (Hasegawa et al., 2013), suggesting that for this kind of complex the size of the lanthanoid ion has a poor influence in the stoichiometry of the isolated compound.

As a summary, comparison of the results described herein with those previously described for Cy_3_PO (Chen et al., 2016, 2017) seems to indicate that the lower cone angle for triphenylphosphine compared with tricyclohexylphosphine prevents the isolation of pentagonal bipyramidal complexes of triphenylphosphine oxide with dysprosium or terbium. In the same way, hexafluoroacetylacetone allows coordination of two phosphine oxide ligands, leading to coordination number of 8 for the lanthanoid atom while the more sterically hindered 2,2,6,6-tetramethylheptane-3,5-dione only allows linking one phosphine ligand, leading to coordination number 7 (Yanagisawa et al., 2017). Therefore, this study clearly shows that not only the cone angle of the phosphine oxides but also the volume of the auxiliary ligands are fundamental factors in controlling the coordination number in this type of complex, the salt:oxide metal ratio, and even the size of the lanthanoid ion, playing secondary roles.

All the compounds described herein were fully characterized by analytical techniques, IR spectroscopy and by single crystal X-ray diffraction studies, except **3**. In addition, direct and alternating current magnetic measurements were recorded for all the compounds.

The IR spectra of all the phosphine oxide complexes show strong bands in the range 1119–1153 cm^−1^, which can be assigned to the P = O stretching vibration (Bowden et al., 2012). For complexes **1**, **2**, and **3** there are two bands in this range, indicating the existence of two types of non-equivalent phosphine oxide. Nevertheless, complexes **6**-**8** present just one band at *ca*. 1135 cm^−1^, which suggest that all the phosphine oxide ligands present in the compounds are equivalent.

Complexes **4** and **5**·1.25H_2_O show strong bands about 900 cm^−1^, which can be assigned to As = O vibrations (Levason et al., 2001). In **4** two bands are observable, also suggesting two non-equivalent arsine oxide ligands, while **5**·1.25H_2_O only shows an As = O band, in agreement with the equivalence of the four arsine oxide donors present in the complex.

In addition, the synthetized complexes also present bands related to the auxiliary nitrate, triflate or hexafluoroacetylacetonate ligands. Thus, complexes **1**-**4** show a strong band at *ca*. 1305 cm^−1^, assigned to the ν_1_(N-O) vibration of the nitrate ligand, acting as bidentate (Bowden et al., 2012). Complex **6** show many bands (633, 1020, 1262, 1156, and 1220 cm^−1^) related to S-O or C-F vibrations, which suggest the presence of triflate (Johnston and Shiver, 1993). Finally, the IR spectra of **7** and **8** present strong bands at 1652 cm^−1^, assigned to the stretching frequency of the C = O group, and at 1250 and 1161, in agreement with vibrations of the CF_3_ and C-H moieties, all of which clearly suggest the presence of the hexafluoroacetylacetonate ligand (Richardson et al., 1968).

### X-ray diffraction studies

#### [Tb(NO_3_)_3_(Ph_3_PO)_3_]·CH_3_CN (1·CH_3_CN) and [Dy(NO_3_)_3_(Ph_3_PO)_3_]·CH_3_CN (2·CH_3_CN)

**Figure 2:**
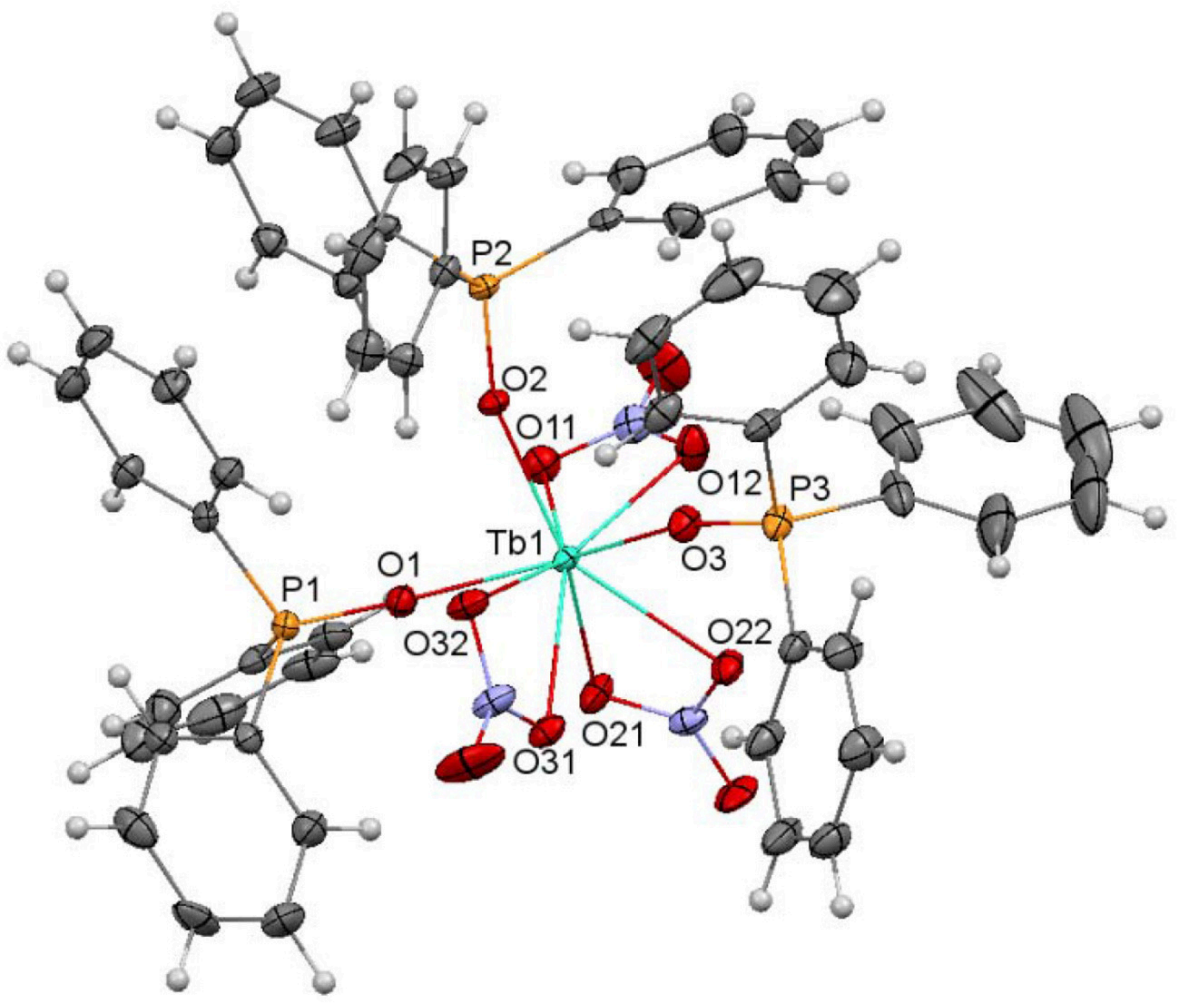
Ellipsoids diagram (50% probability) for **1**. Only the Tb, P, and donor O-atoms have been labeled, for clarity. Color code: Tb, light blue; C, gray; H, light gray; N, dark blue; P, orange; O, red.

The crystal structures of **1**·CH_3_CN and **2**·CH_3_CN are very similar and they will be discussed together. The unit cell of each complex contains neutral [M(NO_3_)_3_(Ph_3_PO)_3_] (M = Tb or Dy) molecules, in addition to acetonitrile as solvate. Ellipsoid diagrams for **1** and **2** are shown in Figure 2, Figure S1, respectively, and main distances and angles in Table S2.

Both complexes crystallize in the *P2*_1_*/c* group, with no symmetry elements relating the different ligands.

**Figure 3:**
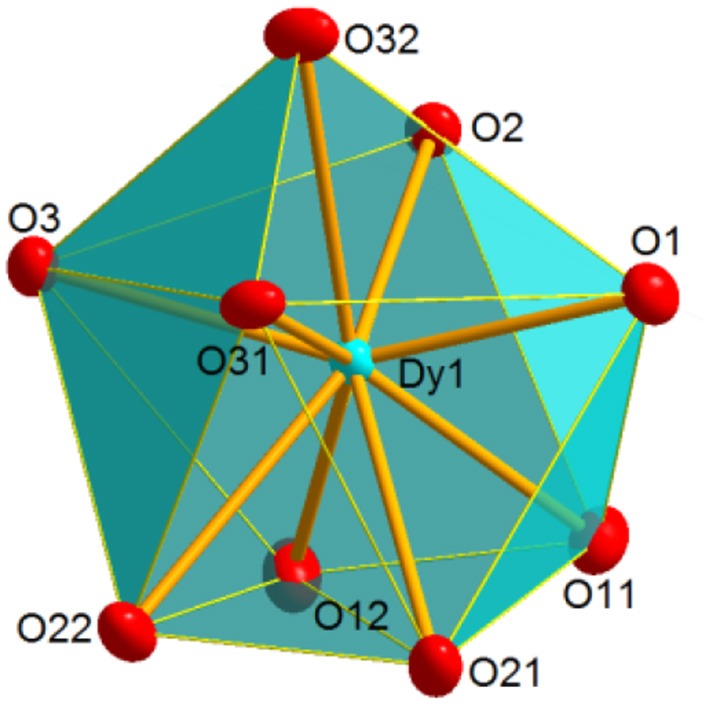
Coordination polyhedron for Dy in **2**, illustrating the distorted spherical capped square antiprism geometry shown by the metal ion in **1** and **2**.

In the [M(NO_3_)_3_(Ph_3_PO)_3_] molecules, three phosphine oxide monodentate ligands and three bidentate chelate nitrate donors complete the coordination sphere of the metal ions. Thus, in both complexes, the lanthanoid ion is in an O_9_ environment. Calculations of the degree of distortion of the LnO_9_ core with respect to a perfect nine-vertex polyhedron using the SHAPE software (Llunell et al., 2005, 2010; Ruiz-Martínez et al., 2008) lead to shape measurements between spherical capped square antiprism and muffin-like, but closer to spherical capped square antiprism (Figure 3, Table S3). In these polyhedra, all the distances and angles are within their usual range for lanthanoid complexes with phosphine oxide and nitrate donors (Bowden et al., 2011), showing M-O_Ph3PO_ distances notably shorter than the M-O_nitrate_ ones (Table S2).

#### [Dy(NO_3_)_3_(EtOH)(Ph_3_AsO)_2_] (4)

**Figure 4:**
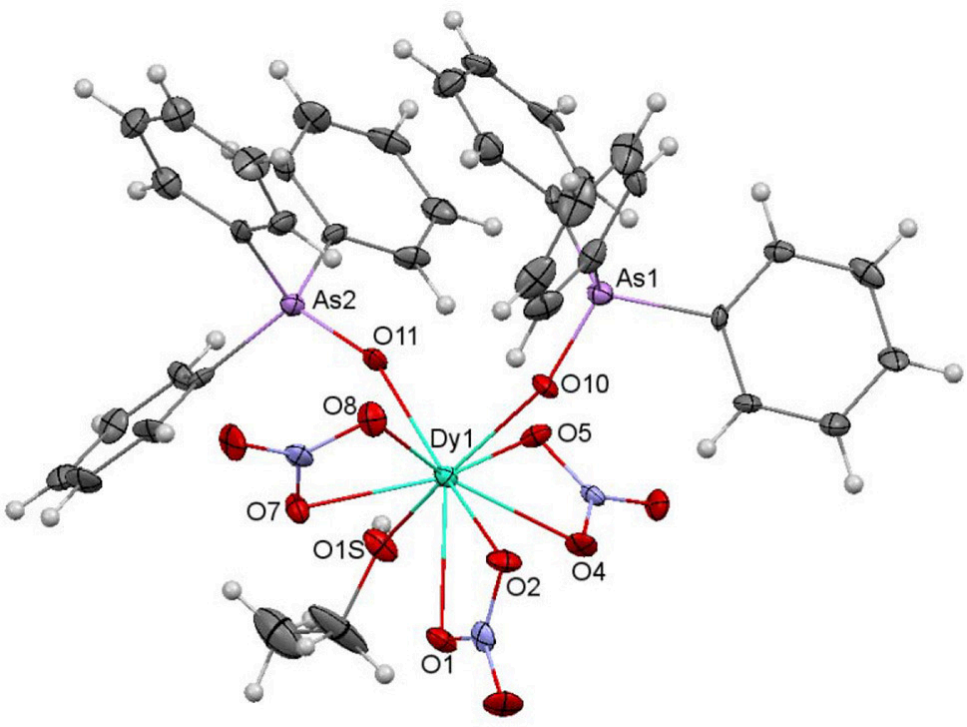
Ellipsoids diagram (50% probability) for **4**. Only the Dy, As, and donor O-atoms have been labeled, for clarity. Color code: Dy, light blue; As, violet; C, gray; H, light gray; N, dark blue; O, red.

An ellipsoid diagram for **4** is shown in Figure 4 and main bond distances and angles in Table S4.

The crystal structure of **4** resembles that of **2**. Besides, it should be noted that **4** is isomorphous with the previously described and crystallographically characterized [La(NO_3_)_3_(EtOH)(Ph_3_AsO)_2_] complex (Levason et al., 2001). Thus, three bidentate chelate nitrate ligands, two monodentate Ph_3_AsO donors and an ethanol molecule fill the O_9_ coordination sphere of the dysprosium ion. SHAPE calculations indicate that the geometry about the metal ion is also between spherical capped square antiprism and muffin-like, as in **1** and **2**, but in this case the muffin-like disposition seems to be a bit more stable (Table S2). All the distances and angles in this complex are within the usual range, but it should be noted that the Dy-O_arsine_ _oxide_ distances are a bit shorter than the corresponding Dy-O_phospine_ _oxide_ ones in **2**, and this can be a consequence of the smaller electronegativity of arsenic with respect to phosphorous, and, therefore, to the greater donor strength of the arsine oxide, given that the geometry of both complexes is very similar.

**Figure 5:**
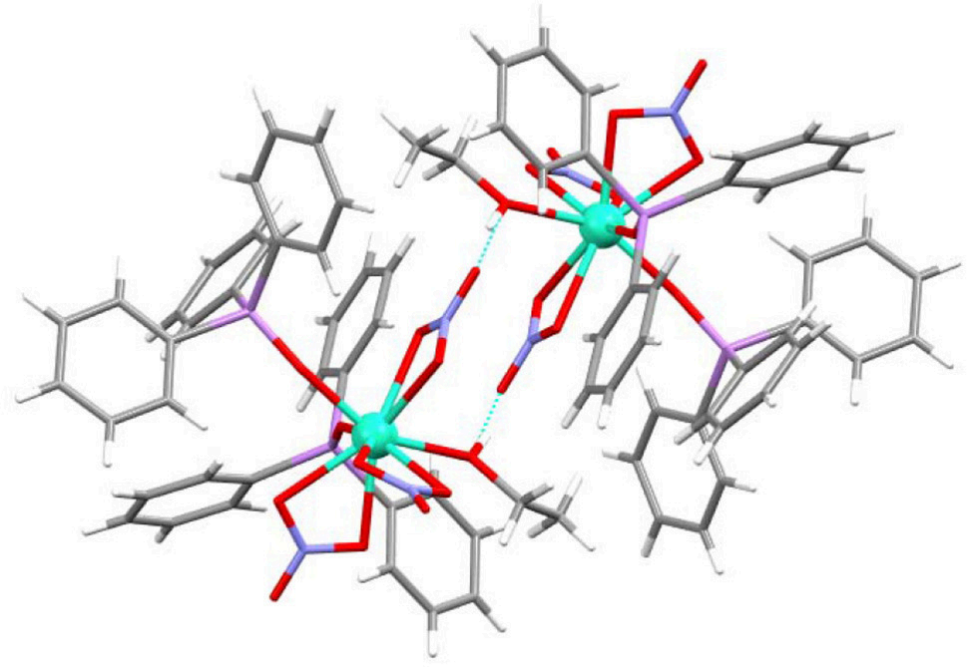
Hydrogen bond for **4**, showing the pseudodimer.

Finally, it is worth of mention that this complex shows a hydrogen bond between one oxygen atom (O6) of one nitrate donor and the ethanol ligand of a neighboring unit. This hydrogen bond is reciprocal and gives rise to a pseudodimer, with a Dy···Dy distance of 7.1870(9) Å (Figure 5).

#### [DyCl_2_(Ph_3_AsO)_4_]Cl·2.75CH_3_CH_2_OH·1.25H_2_O (5·2.75 CH_3_CH_2_OH·1.25H_2_O)

**Figure 6:**
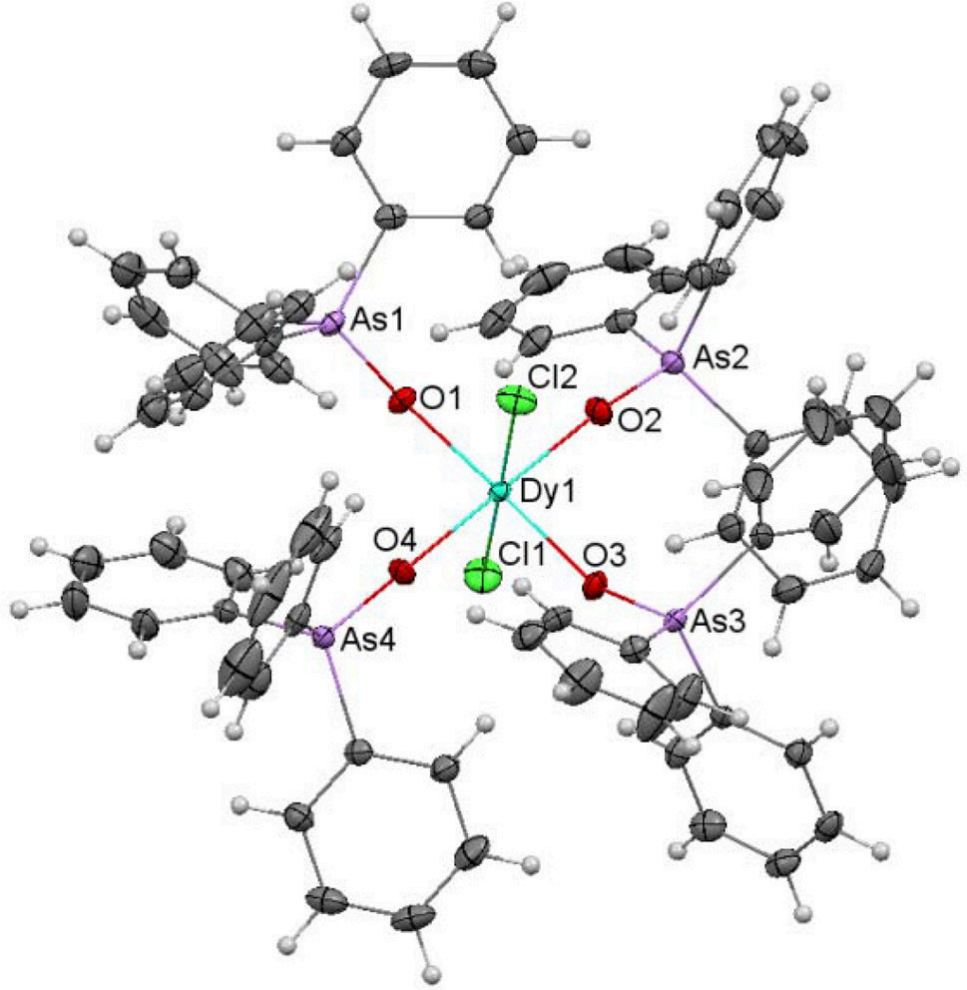
Ellipsoids diagram (50% probability) for [DyCl_2_(Ph_3_AsO)_4_]^+^ in **5**. Only the Dy, As, Cl and donor O-atoms have been labeled, for clarity. Color code: Dy, light blue; As, violet; C, gray; Cl, green; H, light gray; O, red.

An ellipsoid diagram for [Dy(Ph_3_AsO)_4_(Cl)_2_]Cl is shown in Figure 6 and main distances and angles in Table S5.

The asymmetric unit of **5**·2.75CH_3_CH_2_OH·1.25H_2_O contains a [DyCl_2_(Ph_3_AsO)_4_]Cl molecule, and ethanol and water as solvates. The [DyCl_2_(Ph_3_AsO)_4_]^+^ cation shows the Dy(III) ion joined to four Ph_3_AsO ligands through their O-atom, and to two chloride ions. Thus, the dysprosium atom is in an O_4_Cl_2_ environment, with octahedral geometry. In this polyhedron, the chloride ions occupy the axial sites, what produces an elongation of the octahedron.

Although the octahedral geometry is not very common for lanthanide ions, given that they tend to achieve higher coordination numbers, the structure of [DyCl_2_(Ph_3_AsO)_4_]Cl is very similar to that previously described for other [LnX_2_(R_3_PO)_4_]X (X = Cl or Br) compounds (Glazier et al., 2004; Bowden et al., 2010; Platt, 2017), with distances and angles in the usual range, and that do not merit further consideration.

#### [Dy(OTf)_2_(MePh_2_PO)_4_]OTf·THF (6·THF)

The unit cell of **6**·THF contains two crystallographically different but chemically equivalent [Dy(OTf)_2_(MePh_2_PO)_4_]^+^ cations, which will be called **6A** and **6B**, respectively, and CF_3_${\text{SO}}_{3}^{-}$ counterions and THF as solvate. Each [Dy(OTf)_2_(MePh_2_PO)_4_]^+^ cation has an inversion center, located at the dysprosium atom. Besides, one of these [Dy(OTf)_2_(MePh_2_PO)_4_]^+^ cations, the one containing Dy2 (**6B**), has the coordinated triflate anions disordered over two sites (80 and 20% occupancy).

**Figure 7:**
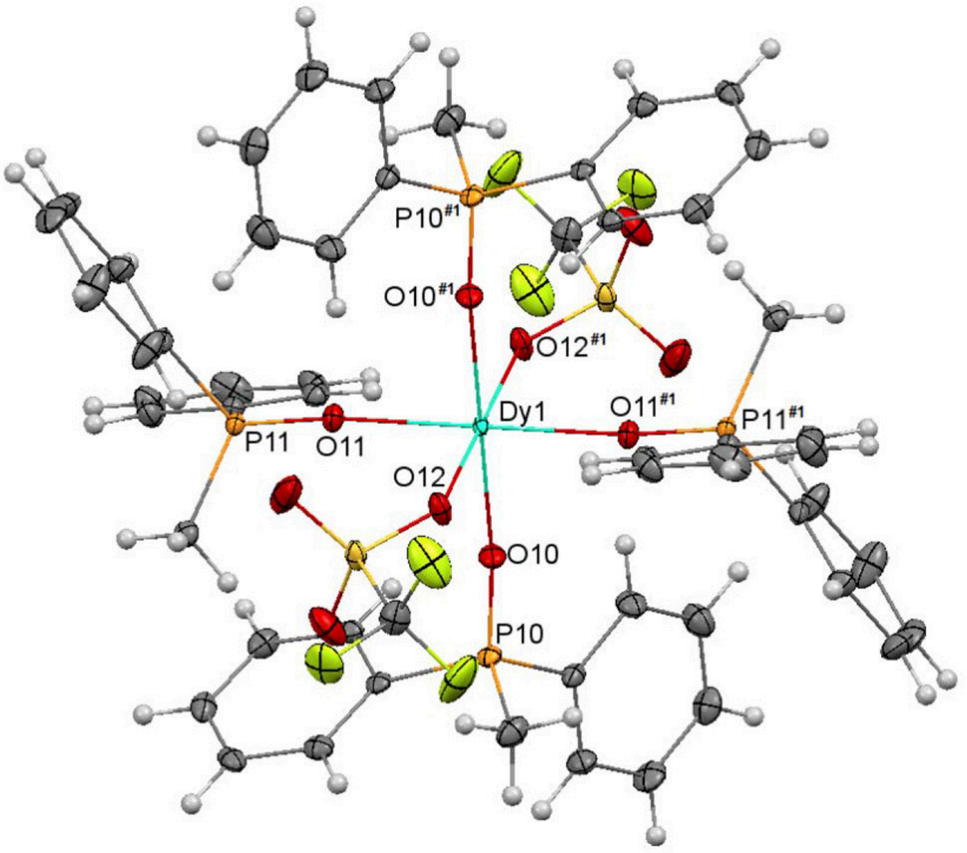
Ellipsoids diagram (50% probability) for [Dy(MePh_2_PO)_4_(OTf)_2_]^+^ in **6A**. Only the Dy, P, and donor O-atoms have been labeled, for clarity. Color code: Dy, light blue; C, gray; F, fluorescent yellow; H, light gray; O, red; P, orange.

An ellipsoid diagram for one of the [Dy(MePh_2_PO)_4_(OTf)_2_]^+^ ions (**6A**) is shown in Figure 7 and main bonds distances and angles in Table S6.

In both [Dy(MePh_2_PO)_4_(OTf)_2_]^+^ cations, the Dy^III^ ions are coordinated to four phosphine oxides and to two triflate ions, which act as monodentate ligands. This gives rise to an O_6_ octahedral environment about the dysprosium center. It should be noted that three lanthanoid complexes of empirical formula [Ln(OTf)_2_(Ph_3_PO)_4_](OTf)(Ln = Ce, Nd, Lu) have been previously reported, and in Ce and Nd complexes one of the triflate ligands acts as bidentate chelate and the other one as monodentate (Fawcett et al., 2002; Berthet et al., 2003), leading to coordination number 7, while in **6** both triflate donors act as monodentate. In fact, the structure of **6** is more similar to that of [Lu(OTf)_2_(Ph_3_PO)_4_](OTf) (Fawcett et al., 2002), where the metal ion is also in an octahedral environment. This seems to be related to the smaller size of Dy^III^ compared with Ce^III^ and Nd^III^ that, in spite of the less sterically hindered phosphine oxide in **6** (MePh_2_PO) respect to the Ce and Nd complexes (Ph_3_PO), cannot allocate seven atoms in its surroundings.

The shortest Dy-O distances in both **6A** and **6B** cations correspond to Dy-O_phosphine_ _oxide_, the weakest bonds being the Dy-O_triflate_ ones, as expected in view of the poor ability of the triflate anions to act as ligands. Thus, the polyhedra are axially elongated. Nevertheless, it should be noted that while in **6A** all the Dy-O_phosphine_ _oxide_ distances are very close, in **6B** the strength of these bonds differs significantly (Dy2-O21 distance of *ca*. 2.19 Å and Dy2-O20 distance of ca. 2.24 Å) and the Dy-O_triflate_ bond is a bit stronger in **6B** than in **6A**, showing a less elongated octahedron.

#### [Tb(hfa)_3_(Ph_3_PO)_2_] (7) and [Dy(hfa)_3_(Ph_3_PO)_2_] (8)

**Figure 8:**
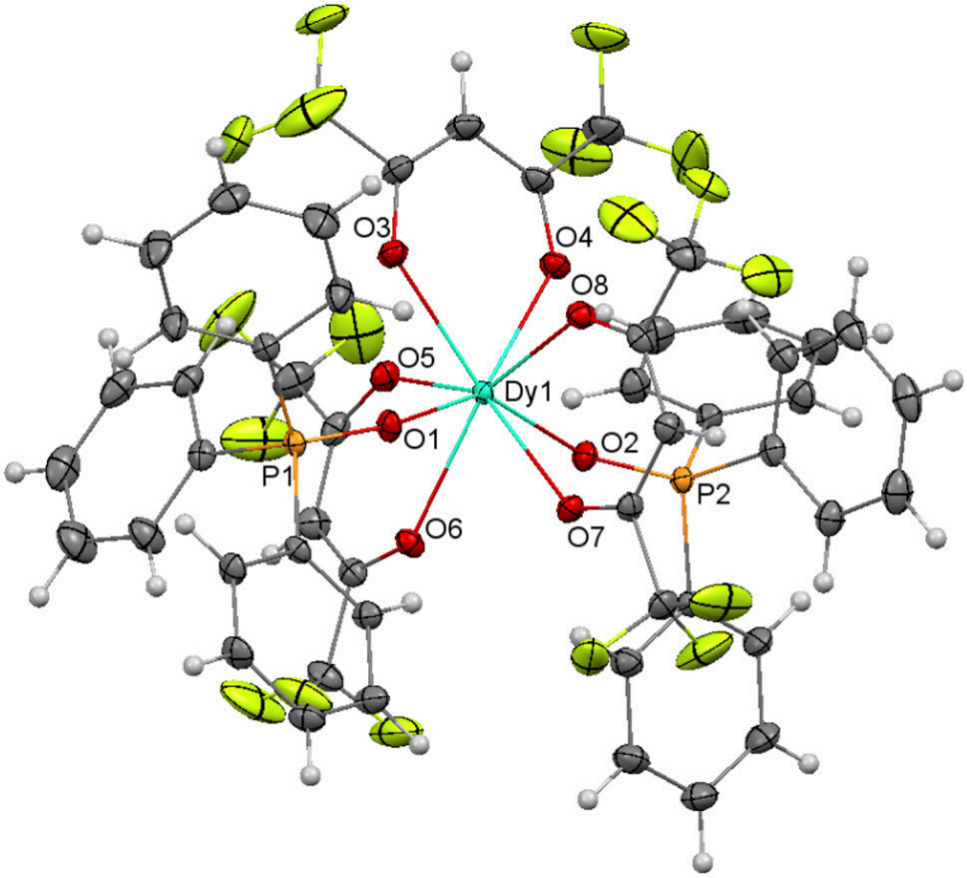
Ellipsoids diagram (50% probability) for **8**. Only the Dy, P, and donor O-atoms have been labeled, for clarity. Color code: Dy, light blue; C, gray; F, fluorescent yellow; H, light gray; O, red; P, orange.

The crystal structures of both complexes are very similar, and they will be discussed together. The main difference between them is that the fluoride atoms of one CF_3_ moiety of one triflate ligand are disordered over two sites (56/44%) in **7**, while this does not occur in **8**. Ellipsoids diagrams for **7** and **8** are shown in Figure S2, Figure 8, respectively, and main bond distances and angles in Table S7.

The structure of both compounds resembles that of [Eu(hfa)_3_(Ph_3_PO)_2_] (Hasegawa et al., 2013). Thus, in both complexes the lanthanoid ion is coordinated to three bidentate chelate hexafluoroacetylacetonate anions and to two phosphine oxides, giving rise to an O_8_ environment. Calculations of the degree of distortion of the MO_8_ environment with respect to an ideal eight-vertex polyhedron with the SHAPE software (Llunell et al., 2005, 2010; Ruiz-Martínez et al., 2008), indicates that the geometry is closer to square antiprism in both cases. In these polyhedra, all the distances and angles are in the expected range and do not merit further consideration.

### Magnetic properties

The direct current (*dc*) magnetic susceptibility studies of all the complexes (**1**-**8**) were recorded under a magnetic field of 1000 Oe in the temperature range 2–300 K. The plots of χ_M_*T vs. T* for the complexes are shown in Figure S3. At room temperature (300 K), the χ_M_*T* products for the terbium complexes **1** and **7** are in the range 11.81–12.37 cm^3^kmol^−1^ while for the dysprosium complexes **2-6** and **8** vary from 14.05 to 14.93 cm^3^kmol^−1^. All these values are similar to the expected ones for one isolated Tb^III^ (4f^8^, ^7^*F*_6_) or Dy^III^ (4f^9^, ^6^*H*_15/2_) ion of 11.82 and 14.17 cm^3^kmol^−1^, respectively.

The experimental χ_M_*T* values remain basically constant (**1**, **2**), decreases very slowly (**6-8**) or decrease a bit quickly (**3**-**5**·1.25H_2_O) upon cooling up to 50 K, and more rapidly below this temperature in all cases. This behavior appears to be mostly owing to the depopulation of the *M*_J_ sublevels of the lanthanoid ion, and possibly to very weak intermolecular interactions, which could be the cause of the more marked decrease in χ_M_*T* at low temperature.

The variation of the magnetization with the field at 2 K for **1**-**8** (Figure S3) are akin in all cases, and shows a quite fast increase of the *M*/Nμ_B_ value at low field, without attaining saturation at 5 T. The *M*/Nμ_B_ value at the largest applied field of 5 T (in the 5.07–5.77 *Ms*/*N*μ_B_ range) is remarkably lower than the predictable one for isolated Tb^III^ or Dy^III^ ions (*Ms*/*N*μ_B_ = *g*_*j*_*J*), which can be ascribed to the crystal-field effects that promote a significant magnetic anisotropy.

Alternating current (*ac*) magnetic susceptibility measurements were also done for microcrystalline samples of compounds **1**-**8**, with the aim of knowing their low-temperature magnetic relaxation behavior. No one of the compounds show out-of-phase *ac* susceptibilities (χ″) peaks at a frequency of 1400 Hz at zero field, indicating the absence of SMM behavior. Nevertheless, it should be noted that, sometimes, application of an external static field (Ruiz et al., 2012), in order to eliminate the mixing of the ground ±*M*s levels, can partially or completely suppress the quantum tunneling of the magnetization (QTM), allowing to observe the slow relaxation process through the real thermally activated energy barrier (*U*). Therefore, this approximation was tested with the aim of checking the quantum tunneling effects.

**Figure 9:**
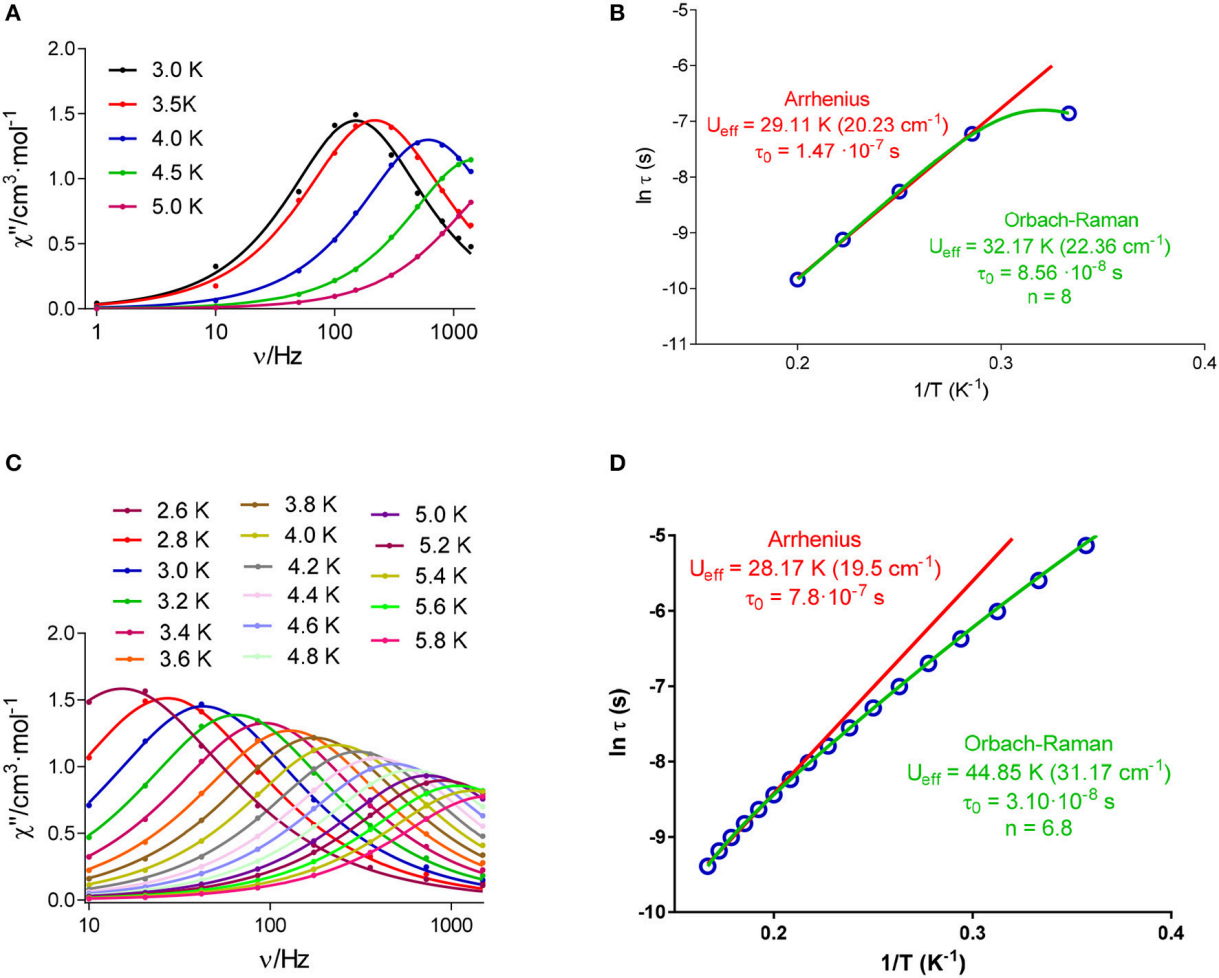
Frequency dependence of out-of-phase component of the *ac* susceptibility (χ”) under a *dc* applied field of 1000 Oe at different temperatures for **2 (A)** and **8 (C)**; and Arrhenius plot for **2 (B)** and **8 (D)**. The solid lines represent the best fit to simple Arrhenius or Orbach-Raman equation.

Accordingly, variable temperature alternating current (*ac*) susceptibilities were measured for all the compounds at 1400 Hz and under a small *dc* field of 1000 Oe. In these conditions, the χ” *vs. T* curve only shows a clear net peak for **2**, **4**, and **8** (Figure S4), but in the case of **4** the peak appears at 2.5 K at a frequency of 1400 Hz. Thus, in view of these results, given that the magnetic response of **4** seems too weak, variable frequency and temperature *ac* susceptibilities were measured only for **2** and **8** with the application of an experimental optimal *dc* field of 1000 Oe. As shown in Figure 9, in these conditions **2** and **8** show frequency and temperature-dependence of the out-of-phase susceptibility, with maxima for χ” below 5 and 5.8 K for **2** and **8**, respectively.

This indicates that both complexes (**2** and **8**) are field-induced single ion magnets. In addition, it should be noticed that Figure 9 reveals that χ” goes to zero below the maxima at low temperature for **2**, which suggest that the fast relaxation of the magnetization by a quantum tunneling mechanism has been completely suppressed by the application of the external magnetic field of 1000 Oe. Nevertheless, this does not occur for **8**, where the QTM does not seem fully suppressed at low temperatures.

According to this, the experimental data for **2** (Figure 9B) were initially fitted to the Arrhenius equation (thermally activated Orbach relaxation process), and the best fit affords a small *U*_*eff*_ barrier of 29.11 K. But the experimental data deviate from the linear Arrhenius plot, which can be due to the simultaneous presence of different relaxation processes.

**Figure 10:**
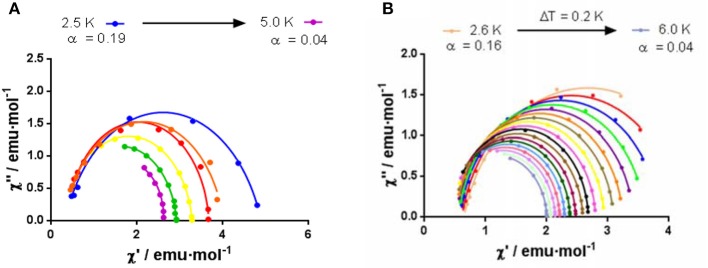
Cole–Cole plot in 1000 Oe *dc* field for **2 (A)** and **8 (B)**.

In keeping with this, the Cole-Cole plot for **2** (Figure 10) between 2.5 and 5 K gives parameters in the range 0.19–0.04, suggesting more than one relaxation process at the lowest temperature. Therefore, the experimental relaxation times were fitted to equation 1, which takes into account contributions from Orbach and Raman processes.

(1) $$\tau^{-1}={\tau_{0}}^{-1}e^{-U_{eff}/k_{B}T}+CT^{n}$$

The best fit with this equation (Figure 9B), which reproduces all the curve, leads to an *U*_*eff*_ barrier of 32.17 K.

In the case of **8**, the best fit of the experimental relaxation times to the Arrhenius equation leads to an *U*_*eff*_ of 28.17 K. However, as in the case of **2**, the experimental relaxation times deviate from linearity (Figure 9), indicating the possible existence of several relaxation processes. The Cole-Cole plot for **8** between 2.6 and 6 K (Figure 10), which renders parameters in the range 0.16–0.04, corroborate the presence of more than one relaxation process, especially in the low-temperature area. In view of this, the experimental data were also fitted to the equation 1. The best fit led to the parameters indicated in Figure 9D. Although for Kramers ion like Dy^III^ an n value equal to 9 is expected (Abragam and Bleaney, 1970), values between 2 and 7 are also realistic when both acoustic and optical phonons are present (Singh and Shrivastava, 1979; Shrivastava, 1983).

At this point it is worth mentioning that the free ion electronic density of the Dy^III^ ion has an oblate shape, which can be stabilized by an axial crystal field leading to an axial anisotropy in the ground state (*M*_*J*_ = ± 15/2). This bistable ground state favors the SMM behavior. The axial crystal field is relatively easy to attain in low-symmetry Dy^III^ complexes, and this is the reason why this type of complexes usually shows slow relaxation of the magnetization and SMM behavior, either at zero applied *dc* field or in the presence of a magnetic field (when a significant QTM is operative).

The non-Kramers Tb^III^ ion is also an oblate ion and therefore also requires an axial crystal field to reach an axial bistable ground state, and then to exhibit SMM behavior. However, to this end a rigorous axial symmetry must be maintained, which is not an easy task. Moreover, non-Kramers ions possess an intrinsic tunneling gap that promotes QTM at zero field. Therefore, the lack of SMM at zero field, and in the presence of a small field for complexes **1** and **7** is not unexpected.

The fact that, among the Dy^III^ complexes, only compounds **2**, **4**, and **8** exhibit slow relaxation of the magnetization can be explained by analyzing the Dy-O distances and their distribution in the Dy^III^ coordination sphere, as these factors play an essential role in dictating the SMM behavior. The required axial crystal field can be reached when the donors atoms with the largest electron densities (generally those involved in the shortest Dy-donor distances) are located above and below the equatorial plane, thus diminishing the repulsive interactions between the donor atoms of the ligands and the oblate charge cloud of the Dy^III^ ion. Nevertheless, the crystal field can also be generated when a Dy-donor distance is significantly shorter that the other Dy-donor distances. Taking into account these considerations, complexes **5** and **6**, which exhibit an elongated octahedral geometry, with the four shortest Dy-O distances in the equatorial plane, present an equatorial crystal field. Therefore, the fact that these complexes do not present SMM behavior is not surprising. In the case of **2**, the three Dy-O_phosphine_ _oxide_ distances, with a near T disposition, are significantly shorter than the other ones. Nevertheless, two of them (Dy-O1 and Dy-O2) are slightly but significantly shorter that the other one. This disposition leads to a feeble axial crystal field that favors slow relaxation of the magnetization. The fact that at zero-field this compound does not show any peak in the *ac* measurements can be due to the fast QTM promoted transverse anisotropy, as well as to intermolecular and hyperfine interactions. For complex **4**, the shortest Dy-O distances are also those involving the oxygen atoms of the arsine-oxide ligands. These distances are approximately 0.2 and 0.1 Å shorter than those of the coordinated nitrate anions and ethanol molecule, respectively. This disposition of the shortest Dy-O distances is similar to that of **2** and therefore the appearance of field induced slow relaxation in **4** is not unexpected. Finally, for compound **8**, the two shortest Dy-O bond distances are those involving the Ph_3_PO ligands, which are at opposite sides of the Dy^III^ ions, with a O1-Dy-O2 angle of 143.63(4)°. This distribution of the phosphine ligands around the Dy^III^ ions generates a clear axial crystal field that is responsible of the SMM behavior observed for this compound.

## Conclusions

The stoichiometry of the Dy and Tb complexes derived from R_3_XO (R_3_ = Ph or MePh_2_, X = P or As) does not seem to rely on the nature of the X atom, nor in the molar ratio of the lanthanoid salt:R_3_XO employed. Nevertheless, this stoichiometry highly depends on the nature of the lanthanoid salt, as well as on the election of the solvent of the reaction.

Comparison of the results obtained herein with previously published data allows concluding that the cone angle of the phosphine or arsine, as well as the degree of steric hindrance of the auxiliary donors also play an important role in the stoichiometry of the isolated compounds. Thus, in our case, it was not possible to obtain pentagonal bipyramidal complexes. In this way, nona-coordinated complexes of different stoichiometry ([M(NO_3_)_3_(Ph_3_PO)_3_] (M = Tb, **1**; Dy, **2**) or [Dy(NO_3_)_3_(EtOH)(Ph_3_XO)_2_] (X = P, **3**; As, **4**) are isolated by mixing the lanthanoid salt and Ph_3_XO in 1:2 molar ratio, just varying the solvent of the reaction. Hexa-coordinated complexes [DyCl_2_(Ph_3_AsO)_4_]Cl (**5**) and [Dy(OTf)_2_(MePh_2_PO)_4_]OTf (**6**; OTf = triflate) are also obtained from 1:2 molar ratios of salt and phosphine oxide, while octa-coordinated [M(hfa)_3_(Ph_3_PO)_2_] (M = Tb, **7**; Dy, **8**) compounds are formed when the lanthanoid precursor and the phosphine oxide are mixed in 1:1 molar ratio.

The magnetic characterization of all the complexes show that only **2**, **4**, and **8** present slow relaxation of the magnetization under an external *dc* field of 1000 Oe. This indicates that, between oblate Tb^III^ and Dy^III^, the Kramers dysprosium ion increases the effective barrier for reversal of the magnetization with respect to non-Kramers terbium ion, as frequently observed, provided that the relaxation of the magnetization is seen for the dysprosium complexes **2**, **4**, and **8** but not for the homologous terbium compounds **1** and **7**. Besides, the arsine oxide also favors the SMM behavior compared with the phosphine oxide, given that **4** shows slow relaxation of the magnetization at low temperature (< 2.5 K) while **3** lacks this relaxation. In addition, the replacement of a phosphine oxide by an ethanol molecule decreases the ability of the complex to act as a SIM, since **2** is a field induced SIM, with an *U*_*eff*_ barrier of 32.17 K, while **3** does not show any evidence of SMM behavior. Furthermore, [Dy(hfa)_3_(Ph_3_PO)_2_] is also a weak induced SIM, with *U*_*eff*_ of 44.85 K. The field induced slow relaxation of the magnetization in complexes **2**, **4**, and **8**, as well as the absence of slow relaxation in complexes **5** and **6** has been justified by using the simple prolate-oblate electrostatic model and the distribution of the shortest Dy-O distances in the Dy^III^ coordination sphere.

## Author contributions

JC-V and JS-M did the experimental work to prepare, crystallize and characterize the samples. AMG-D carried out the XRD analyses. JMH and EC made the magnetic measurements, interpreted some of the magnetic results and participate in the writing. MF designed and supervised the work, interpreted some of the magnetic data and wrote the draft.

### Conflict of interest statement

The authors declare that the research was conducted in the absence of any commercial or financial relationships that could be construed as a potential conflict of interest.
