# Supplementary material for: Field-Induced Single Molecule Magnets of Phosphine- and Arsine-Oxides
Source: Front Chem. 2018 Sep 12;6:420. doi: 10.3389/fchem.2018.00420 (PMC6143903; doi:10.3389/fchem.2018.00420)
Supplement: Supplementary file 1 [file Data_Sheet_1.pdf]

Supplementary Material

Field-induced Single Molecule Magnets of Phosphine- and Arsine-oxides

Matilde Fondo,[1*] Julio Corredoira-Vázquez,[1] Ana M. García-Deibe,[1] Jesús Sanmartín-Matalobos,[1] Juan Manuel Herrera,[2] Enrique Colacio[2]

*** Correspondence:** matilde.fondo@usc.es

Table S1. Crystal data and structure refinement for **1**·CH_3CN, **2**·CH_3CN, **4**, **5**·2.75EtOH·1.25H_2O, **6**·THF, **7** and **8**.

	**1**·CH_3CN	**2**·CH_3CN	**4**	**5**·2.75EtOH·1.25H_2O
Formula	$C_{56}H_{48}N_4O_{12}P_3Tb$	$C_{56}H_{48}DyN_4O_{12}P_3$	$C_{38}H_{36}As_2DyN_3O_{12}$	$C_{77.50}H_{78.50}As_4Cl_3DyO_8$
M.W.	1220.81	1224.39	1039.04	1706.43
Crystal system	Monoclinic	Monoclinic	Monoclinic	Triclinic
Space group	*P2₁/c* → $P2_1/c$	$P2_1/c$	$P2_1/n$	*P-1*
Wavelength (Å)	0.71073	0.71073	0.71073	0.71073
Crystal size (mm^3)	0.36 × 0.33 × 0.07	0.44 x 0.14 x 0.12	0.30 × 0.22 × 0.02	0.40 × 0.14 × 0.10
T (K)	100(2)	100(2)	100(2)	100(2)
a (Å)	11.1117(9)	11.0948(3)	17.205(2)	13.3256(7)
b (Å)	11.3158(9)	11.3316(3)	10.8291(16)	15.0474(8)
c (Å)	43.527(3)	43.4195(12)	21.486(3)	19.5940(8)
α (º)	90	90	90	77.555(3)
β (º)	96.3180(10)	96.266(2)	105.657(6)	81.915(3)
γ (º)	90	90	90	84.088(3)
Volume ($Å^3$)	5439.7(7)	5426.2(3)	3854.7(9)	3787.7(3)
Z	4	4	4	2
Absorp. Coef. (mm^{-1})	1.454	1.531	3.710	2.878
Reflections collected	61237	71229	36881	121087
Independent reflections	11070 [R_{int} = 0.0430]	13441 [R_{int} = 0.0518]	6953 [R_{int} = 0.1352]	18783 [R_{int} = 0.0405]
Data / restraints / param.	11070 / 0 / 686	13441 / 0 / 686	6953 / 0 / 506	18783 / 0 / 858
Final R indices [I>2σ(I)]	R_1 = 0.0405	R_1 = 0.0330	R_1 = 0.0558	R_1 = 0.0328
	wR_2 = 0.0798	wR_2 = 0.0616	wR_2 = 0.0876	wR_2 = 0.0654
R indices (all data)	R_1 = 0.0483	R_1 = 0.0445	R_1 = 0.1388	R_1 = 0.0480
	wR_2 = 0.0818	wR_2 = 0.0649	wR_2 = 0.1074	wR_2 = 0.0693

Cont. Table S1. Crystal data and structure refinement for **1**·CH_3CN, **2**·CH_3CN, **4**, **5**·2.75EtOH·1.25H_2O, **6**·THF, **7** and **8**.

	**6**·THF	**7**	**8**
Formula	$C_{59}H_{60}DyF_9O_{14}P_4S_3$	$C_{51}H_{33}F_{18}O_8P_2Tb$	$C_{51}H_{33}DyF_{18}O_8P_2$
M.W.	1546.63	1336.63	1340.21
Crystal system	Triclinic	Monoclinic	Monoclinic
Space group	*P-1*	$P2_1/c$	$P2_1/n$
Wavelength (Å)	0.71073	0.71073	0.71073
Crystal size (mm^3)	0.37 × 0.26 × 0.08	0.40 × 0.39 × 0.20	0.45 × 0.42 × 0.26
T (K)	100(2)	100(2)	100(2)
a (Å)	11.835(2)	12.7514(5)	16.9928(11)
b (Å)	14.920(3)	13.4304(4)	15.3504(10)
c (Å)	20.529(4)	30.2548(11)	20.5689(14)
α (º)	91.857(12)	90	90
β (º)	106.143(11)	91.655(3)	93.986(2)
γ (º)	110.123(11)	90	90
Volume ($Å^3$)	3236.3(10)	5179.2(3)	5352.3(6)
Z	2	4	4
Absorp. Coef. (mm^{-1})	1.438	1.546	1.570
Reflections collected	74060	124146	835229
Independent reflections	15416 [R_{int} = 0.0415]	17264 [R_{int} = 0.0809]	29481 [R_{int} = 0.0904]
Data / restraints / param.	15416 / 0 / 843	17264 / 72 / 749	29481 / 0 / 721
Final R indices [$I>2\sigma(I)$]	R_1 = 0.0297	R_1 = 0.0380	R_1 = 0.0335
	wR_2 = 0.0611	wR_2 = 0.0692	wR_2 = 0.0698
R indices (all data)	R_1 = 0.0402	R_1 = 0.0706	R_1 = 0.0478
	wR_2 = 0.0651	wR_2 = 0.0762	wR_2 = 0.0742

Table S2. Main bond distances (Å) and angles (º) for **1**·CH_3CN and **2**·CH_3CN.

	**1**·CH_3CN	**2**·CH_3CN
M1-O1	2.267(3)	2.2601(17)
M1-O2	2.280(2)	2.2687(16)
M1-O3	2.316(3)	2.3042(18)
M1-O11	2.499(3)	2.4880(18)
M1-O12	2.446(3)	2.4343(18)
M1-O21	2.482(3)	2.4675(18)
M1-O22	2.496(3)	2.4865(17)
M1-O31	2.443(3)	2.4306(17)
M1-O32	2.531(3)	2.5188(18)
O1-M1-O3	150.65(10)	150.41(6)
O21-M1-O22	51.28(9)	51.56(6)

Table S3. Continuous Shape measures calculations for complexes **1**·CH_3CN, **2**·CH_3CN, **4**, **5**·2.75EtOH·1.25H_2O, **6**·THF, **7** and **8**.

Geometries Coordination number 8

ETBPY-8	13	D3h	Elongated trigonal bipyramid
TT-8	12	Td	Triakis tetrahedron
JSD-8	11	D2d	Snub diphenoid J84
BTPR-8	10	C2v	Biaugmented trigonal prism
JBTPR-8	9	C2v	Biaugmented trigonal prism J50
JETBPY-8	8	D3h	Johnson elongated triangular bipyramid J14
JGBF-8	7	D2d	Johnson gyrobifastigium J26
TDD-8	6	D2d	Triangular dodecahedron
SAPR-8	5	D4d	Square antiprism
CU-8	4	Oh	Cube
HBPY-8	3	D6h	Hexagonal bipyramid
HPY-8	2	C7v	Heptagonal pyramid
OP-8	1	D8h	Octagon

Geometries Coordination number 9

MFF-9	13	Cs	Muffin
HH-9	12	C2v	Hula-hoop
JTDIC-9	11	C3v	Tridiminished icosahedron J63
TCTPR-9	10	D3h	Spherical tricapped trigonal prism
JTCTPR-9	9	D3h	Tricapped trigonal prism J51
CSAPR-9	8	C4v	Spherical capped square antiprism
JCSAPR-9	7	C4v	Capped square antiprism J10
CCU-9	6	C4v	Spherical-relaxed capped cube
JCCU-9	5	C4v	Capped cube J8
JTC-9	4	C3v	Johnson triangular cupola J3
HBPY-9	3	D7h	Heptagonal bipyramid
OPY-9	2	C8v	Octagonal pyramid
EP-9	1	D9h	Enneagon

[Tb(NO_3)$_3$(TPPO)$_3$]·CH_3CN (1·CH_3CN)

Structure [ML9]	**MFF-9**	HH-9	JTDIC-9	TCTPR-9	JTCTPR-9	**CSAPR-9**
	**2.179**,	9.741,	11.916,	2.693,	3.249,	**2.130**

JCSAPR-9	CCU-9	JCCU-9	JTC-9	HBPY-9	OPY-9	EP-9
2.891,	8.872,	10.294,	14.262,	17.394,	22.609,	33.748

[Dy(NO_3)$_3$(TPPO)$_3$]·CH_3CN (2·CH_3CN)

Structure [ML9]	**MFF-9**	HH-9	JTDIC-9	TCTPR-9	JTCTPR-9	**CSAPR-9**
	**2.144**,	9.783,	11.950,	2.636,	3.178,	**2.090**

JCSAPR-9	CCU-9	JCCU-9	JTC-9	HBPY-9	OPY-9	EP-9
2.851,	8.912,	10.320,	14.243,	17.433,	22.605,	33.698

[Dy(EtOH)(NO3)3(TPAsO)2] (4)

Structure [ML9]	**MFF-9**	HH-9	JTDIC-9	TCTPR-9	JTCTPR-9	**CSAPR-9**
	**1.987**,	10.817,	11.769,	2.526,	3.288,	**2.008**

JCSAPR-9	CCU-9	JCCU-9	JTC-9	HBPY-9	OPY-9	EP-9
2.783,	9.391,	10.490,	14.624,	17.795,	22.105,	34.388

[Tb(hfacac)3(TPPO)2] (7)

Structure [ML8]	ETBPY-8	TT-8	JSD-8	**BTPR-8**	JBTPR-8	JETBPY-8
	23.144,	11.366,	3.594,	**1.389,**	1.900,	27.755

JGBF-8	TDD-8	**SAPR-8**	CU-8	HBPY-8	HPY-8	OP-8
13.290,	1.703,	**0.580**,	10.531,	15.686,	24.068,	28.126

[Dy(hfacac)3(TPPO)2] (8)

Structure [ML8]	ETBPY-8	TT-8	JSD-8	BTPR-8	JBTPR-8	JETBPY-8
	24.815,	11.217,	2.862,	1.555,	1.947,	28.410

JGBF-8	**TDD-8**	**SAPR-8**	CU-8	HBPY-8	HPY-8	OP-8
14.039,	**1.067**,	**0.822**,	10.674,	16.339,	23.293,	30.043

Table S4. Main bond distances (Å) and angles (º) for **4**.

Dy1-O1	2.517(5)	Dy1-O2	2.463(5)
Dy1-O4	2.456(5)	Dy1-O5	2.523(5)
Dy1-O7	2.444(5)	Dy1-O8	2.490(5)
Dy1-O10	2.223(5)	Dy1-O11	2.253(5)
Dy1-O1S	2.387(5)		
O11-Dy1-O2	151.55(19)	O4-Dy1-O5	51.34(17)

Table S5. Main bond distances (Å) and angles (º) for **5**·2.75CH3CH2OH·1.25H2O

Dy1-O1	2.2215(17)	Dy1-O3	2.2235(17)
Dy1-O2	2.2012(18)	Dy1-O4	2.1911(18)
Dy1-Cl1	2.6793(7)	Dy1-Cl2	2.6452(6)
O1-Dy1-O3	178.25(7)	Cl2-Dy1-Cl1	176.76(2)
O4-Dy1-O2	179.51(7)		

Table S6. Main bond distances (Å) and angles (º) for **6**·THF

**6A**		**6B**	
Dy1-O10	2.2082(15)	Dy2-O20	2.2470(15)
Dy1-O11	2.2128(14)	Dy2-O21	2.1938(15)
Dy1-O12	2.3152(15)	Dy2-O22	2.2743(15)
O10#1-Dy1-O10	180.0	O20-Dy2-O20#2	180.00(3)
O11#1-Dy1-O11	180.0	O21-Dy2-O21#2	180.00(8)
O12#1-Dy1-O12	180.00(5)	O22#2-Dy2-O22	180.0

Table S7. Main bond distances (Å) and angles (º) for **7** and **8**

	**7**	**8**
M1-O1	2.2962(15)	2.2550(11)
M1-O2	2.3008(15)	2.2587(11)
M1-O3	2.3561(16)	2.3892(11)
M1-O4	2.3919(16)	2.3868(11)
M1-O5	2.4002(17)	2.3293(11)
M1-O6	2.3832(16)	2.4183(11)
M1-O7	2.3861(16)	2.3910(11)
M1-O8	2.3861(1-5)	2.3334(11)
O7-Tb1-O8/	71.16(5)	
O5-Dy1-O6		71.66(4)
O3-Tb1-O6/	148.75(6)	
O5-Dy1-O8		145.81(4)

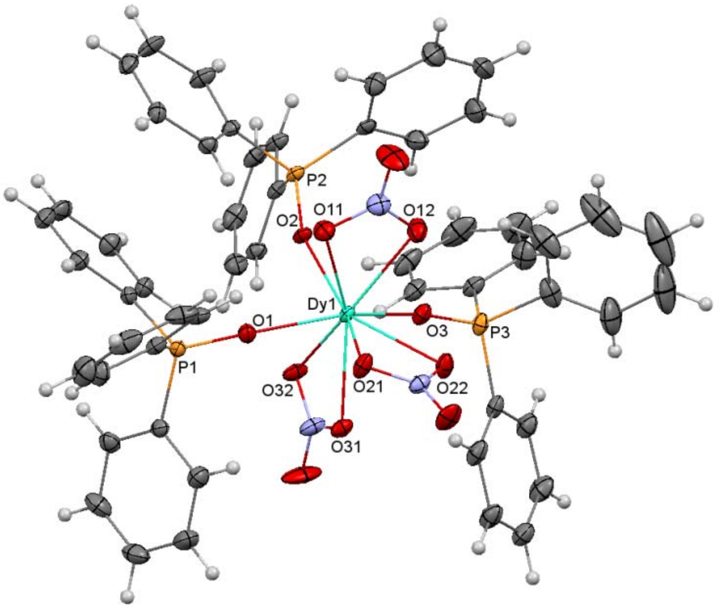

Figure S1. Ellipsoids diagram (50% probability) for **2.** Only the Dy, P, and donor O-atoms have been labelled, for clarity. Color code: Dy: light blue, C: grey, N: dark blue, H: light grey, O: red, P: orange.

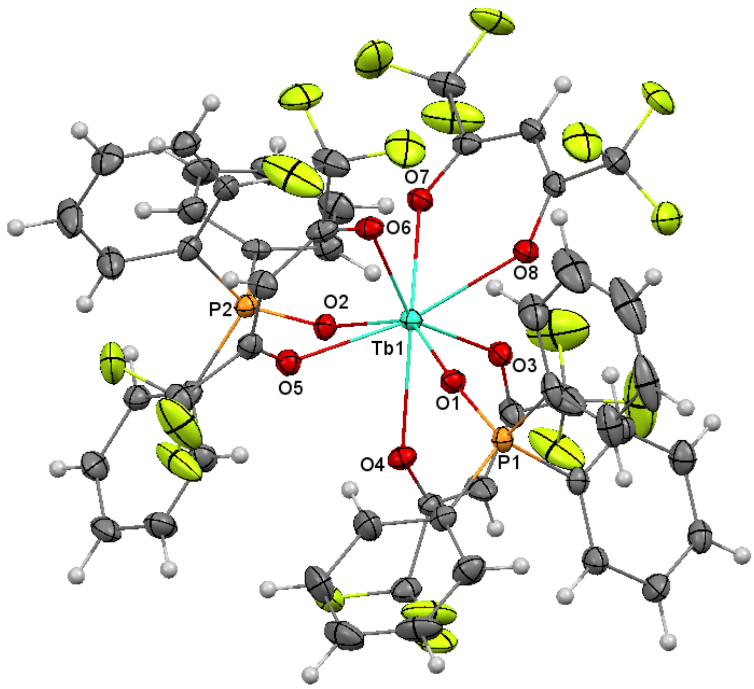

Figure S2. Ellipsoids diagram (50% probability) for **7.** Only the Tb, P, and donor O-atoms have been labelled, for clarity. Color code: Tb: light blue, C: grey, F: fluorescent yellow, H: light grey, O: red, P: orange.

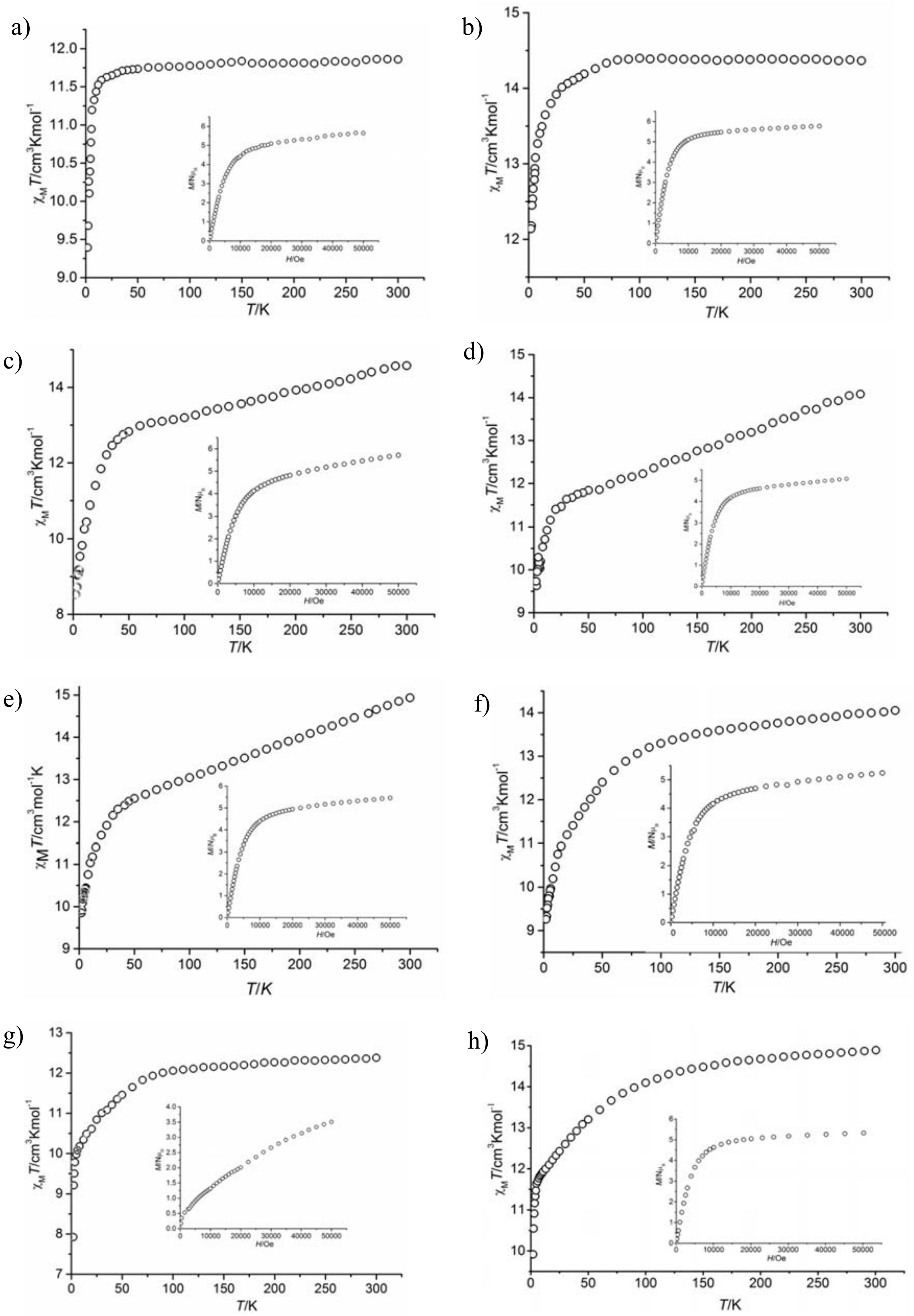

Fig. S3. $\chi_M T$ *vs* *T* for **1** (a), **2** (b), **3** (c), **4** (d), **5**·1.25H_2O (e), **6**·THF (f), **7** (g) and **8** (h). Inset: *M*/Nμ_B *vs* *H*.

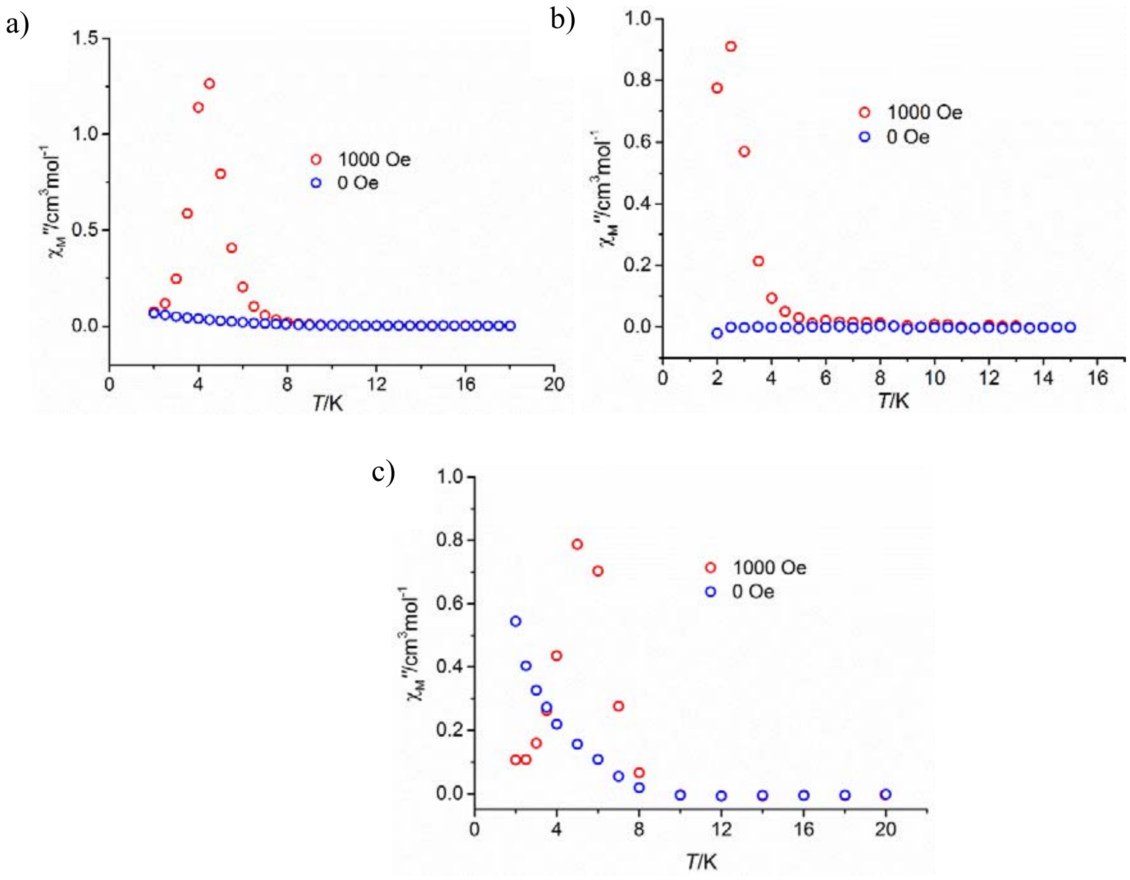

Fig. S4. Temperature dependence of out-of-phase component of the *ac* susceptibility under an applied *dc* field of 0 and 1000 Oe at 1400 Hz for **2** (a), **4** (b) and **8** (c).
